# Experimental Validation of Gaussian Process-Based Air-to-Ground Communication Quality Prediction in Urban Environments

**DOI:** 10.3390/s19143221

**Published:** 2019-07-22

**Authors:** Pawel Ladosz, Jongyun Kim, Hyondong Oh, Wen-Hua Chen

**Affiliations:** 1 Department of Aeronautical and Automotive Engineering, Loughborough University, Loughborough, Leicestershire LE11 3TU, UK; 2 School of Mechanical, Aerospace and Nuclear Engineering, Ulsan National Institute of Science and Technology, Ulsan 44919, Korea

**Keywords:** unmanned aerial vehicles, communication relay, gaussian process regression, wireless communication model, urban environment

## Abstract

This paper presents a detailed experimental assessment of Gaussian Process (GP) regression for air-to-ground communication channel prediction for relay missions in urban environment. Considering restrictions from outdoor urban flight experiments, a way to simulate complex urban environments at an indoor room scale is introduced. Since water significantly absorbs wireless communication signal, water containers are utilized to replace buildings in a real-world city. To evaluate the performance of the GP-based channel prediction approach, several indoor experiments in an artificial urban environment were conducted. The performance of the GP-based and empirical model-based prediction methods for a relay mission was evaluated by measuring and comparing the communication signal strength at the optimal relay position obtained from each method. The GP-based prediction approach shows an advantage over the model-based one as it provides a reasonable performance without a need for a priori information of the environment (e.g., 3D map of the city and communication model parameters) in dynamic urban environments.

## 1. Introduction

Stable communication for a team of multiple robots plays an important role to succeed the given mission in the complex environments. Particularly, in urban environments, provision of reliable communication is challenging due to obstructions by buildings, inducing significant delays and limited range and bandwidth. To mitigate this issue, relay unmanned aerial vehicles (UAVs) can be utilized to improve the communication performance of networked ground nodes operating in an urban environment.

Reliable communication quality prediction between networked nodes is essential for successful relay missions. In the literature, numerous approaches are developed for wireless communication channel prediction and trajectory planning of relay UAVs. Shin and Gasco divided communication channel prediction approaches into model-based and measurement-based ones [1]. In the model-based approach, the communication strength between nodes is calculated by using a pre-defined model with known parameters dependent on the mission environment [2,3]. Another example of a model-based approach is proposed in [4], where the UAV is used to transfer command controls from the base station to a distant ground node. The perturbation-based iterative algorithm shows similar performance to that of the brute force approach but with less computation time. The main limitation of model-based approaches is a lack of adaptability to deal with changes in the communication environments since significant prior knowledge (e.g., communication model parameters and 3D map of the environment) is required.

In the measurement-based approach, the communication channel model is constructed in real time by collecting signal strength measurements. This approach could be subdivided into the gradient following and the learning methods. In the gradient following methods, the relay UAV gradually improves the communication quality by following the gradient direction of the measured signal strength [5,6]. However, the gradient following method could be trapped into local optima due to unexpected signal strength variations caused by buildings in urban environments. Besides, this method focuses on the control and trajectory planning aspect rather than communication channel prediction of the mission area.

Learning approaches utilize the real communication data to provide a correction to the a priori communication model or to construct a communication map [7,8,9]. In particular, many researchers exploited Gaussian Process (GP), one of popular machine learning techniques, in communication relay problems successfully [9,10,11,12]. The main benefit of GP-based channel prediction comes from its non-parametric nature and accurate prediction ability even with sparse data. Besides, it requires less a priori information about the environment. However, GPs are often expensive to compute and need to be carefully designed in relay missions as communication measurements are usually very noisy.

Since our previous studies [13,14] deal with GP-based approaches only in numerical simulations, they need to be validated in the real-world settings. In this study, the performance of channel quality prediction using the GP in an urban environment was evaluated through several experiments. Real-world tests in an urban environment are difficult to be performed due to flight restrictions in cities. Hence, this study introduced an artificial urban environment in indoor settings to simulate real-world cities at a room scale while keeping intrinsic properties of real-world cities for relay UAV missions. Since water significantly absorbs wireless signal, water containers were used to take the role of buildings in artificial indoor cities. In the relay mission, the relay UAV collects the signal strength data from ground nodes by performing the pre-planned scan flight and builds the communication map of the environment using the GP-based approach. For validation, the actual channel qualities at certain positions were measured and compared with the GP-based predicted values. Besides, the GP method was compared to an empirical model-based approach to highlight the benefit of the GP.

The main contribution of this paper is threefold as:

- The accuracy and consistency of the GP-based channel prediction method was verified by real experiments in an artificial indoor urban environment.
- It is shown that the GP-based method can be run in real-time for urban relay missions.
- It is shown that the GP-based method provides a reasonably good performance with much less information (e.g., no need of the 3D map of the city and communication model parameters) compared to the model-based approach.

The rest of this paper is organized as follows. In Section 2, the scenario and assumptions are presented and a brief introduction to communication network topologies is given. Section 3 introduces two channel quality prediction methods employed in this paper. The experimental setup is explained in Section 4, followed by experimental results performed in four types of indoor environments in Section 5. Conclusions and future work are given in Section 6.

## 2. Preliminaries

### 2.1. Scenario and Assumptions

**Figure 1:**
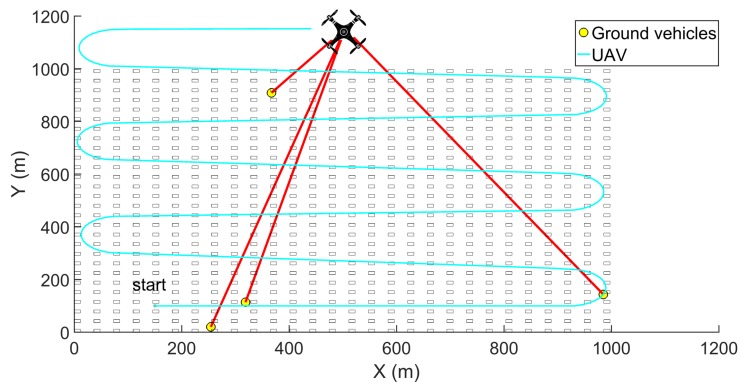
Pattern of UAV scan flight on a sample urban scenario.

This study considered an urban environment in which the UAV performs an aerial communication relay mission for multiple unmanned ground vehicles (UGVs). The relay UAV first scans the area by flying in a pattern, as shown in Figure 1 while collecting the signal strength data. The signal strength value used in this study is received signal strength indicator (RSSI). The UAV computes the communication map and the optimal relay position using the GP-based channel prediction method. The UAV also measures the actual signal strength at the optimal position from the GP and model-based methods to compare their performance.

The assumptions used in this study include: (i) UGVs are stationary and able to transmit their positions to the relay UAV prior to a mission; and (ii) the position and shape of buildings are known for the model-based approach but unknown for the GP-based approach.

### 2.2. Overview of Network Topologies

The network topology determines the structure of connections amongst nodes. In [15], Meador presented a good overview of network topologies. In general, there are four different topologies: ring, star, tree and mesh. The mesh network appears to be the most suitable for the UAV communication relay missions and experiments in this work due to robustness of reconfiguration. The topology is likely to be reconfigured because signal strengths can keep changing, which causes connections between nodes to break or appear. Besides, during a relay mission, the relay node may need to be added and removed in the network. Hence, the mesh network topology was used in this study.

## 3. Communication Quality Prediction

In this section, the empirical model-based and the GP-based approaches for communication quality prediction are presented. The benefits of the GP-based prediction method compared to the model-based one are subsequently discussed.

### 3.1. Empirical Model-Based Approach

To fairly evaluate the communication performance by the GP approach, it is important to compare its performance against existing model-based approaches. Conventional wireless communication models may not be suitable in our experimental setting as the models are generally intended for a much larger scale than the small indoor experiment. Thus, the need for establishing a communication model suitable for our scenarios arises. To develop the communication model, two experiments were performed: (i) to determine the distance-based model for an open space; and (ii) to model the effect of buildings on the wireless communication signal strength. It is worth noting that this might not be the best possible model given the circumstances, however, this model would be better than general multi-purpose models in our experimental setup.

#### 3.1.1. Distance-Based Model

**Figure 2:**
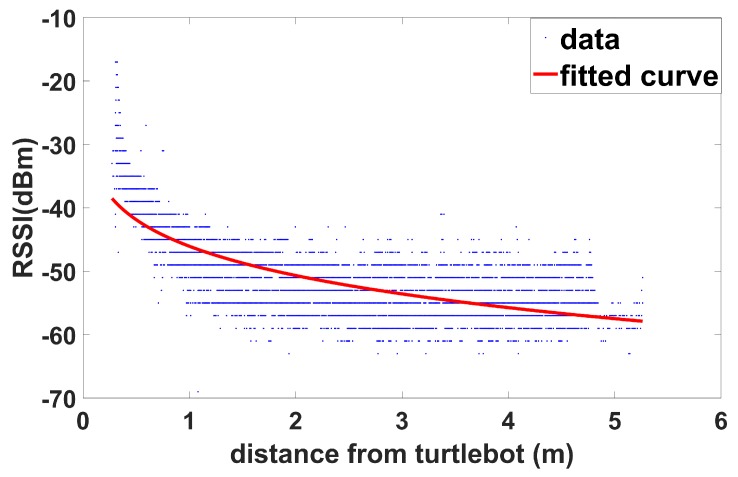
RSSI values with the distance between the UGV (Turtlebot) and the quadrotor UAV. The distance is the ground distance between the UGV and the UAV.

To findthe effect of the distance between the relay UAV and a ground node on the signal strength, the UAV and the ground node are placed in an open indoor space. The collected received signal strength indicator (RSSI) values between two nodes are used to fit the polynomial curve. The signal strength is measured for four directions of the ground vehicle in order to take the directional effects into account. The polynomial fitting yields a distance-based communication model suitable for our experimental area shown as Figure 2, formulated as:

(1) $$W_{di}=-46.05d_{i}^{0.1376}$$

where $W_{di}$ is the LOS signal strength between the node *i* and the UAV and $d_{i}$ is the distance between them.

**Figure 3:**
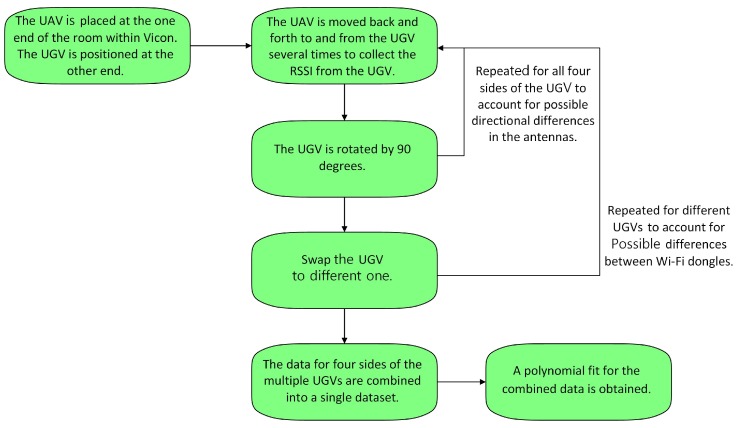
Experimental procedure for calculating the LOS signal strength model.

The detailed procedure is illustrated in Figure 3.

#### 3.1.2. Effects of Buildings

**Figure 4:**
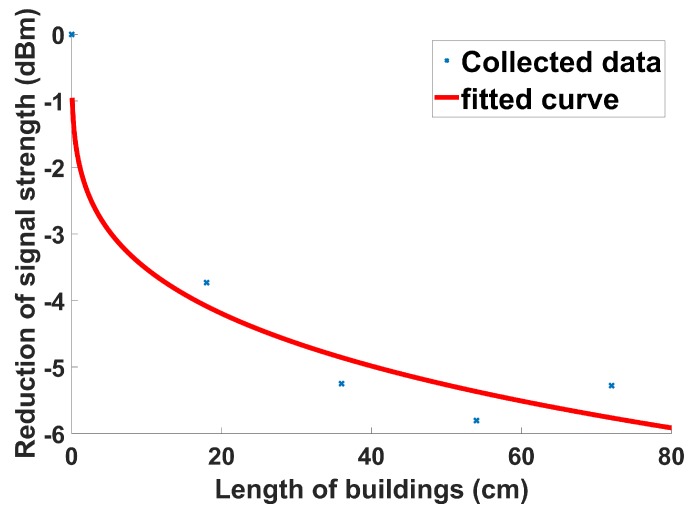
Reduction of signal strength with the length of line-of-sight obstruction in a building.

To consider the effect of building obstruction, buildings are placed between the UAV and the UGV, and then the signal strength is measured and analyzed. One to four buildings (water containers in this study) are considered to collect the data. The dimensions and locations of the buildings are fully known. Figure 4 shows the polynomial curve fitting using the measured data. It can be noted that the fit is nonlinear. The majority of the electromagnetic waves are stopped by initial layers of water containers. Upon entering the next layer of water containers, as there are not many electromagnetic waves, the probability which any of them will meet water particles to be absorbed is significantly smaller. Thus, the overall reduction in the signal strength will be smaller as the length of buildings increases (i.e., as more water containers are involved). The signal reduction $W_{bi}$ by building obstruction is formulated as:

(2) $$W_{bi}=-2.204d_{obs}^{0.2352}+0.2642$$

where $d_{obs}$ is the length of the obstructions in the building.

**Figure 5:**
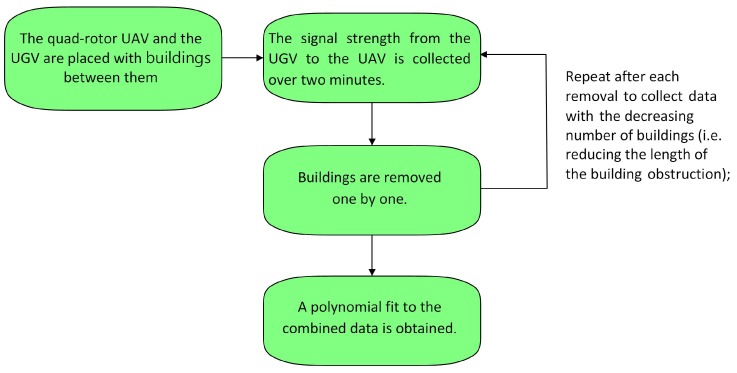
Experimental procedure for calculating the NLOS signal reduction model.

The detailed procedure is illustrated in Figure 5.

By combining above two models, the empirical model-based channel prediction ($W_{i}$) between the UAV and the ground node (i.e., UGV) can be expressed as:

(3) $$W_{i}=W_{di}-W_{bi}.$$

It could be worthwhile reiterating that this empirical communication model is established only for comparison with the GP approach as a benchmark method.

### 3.2. Gaussian Process-Based Approach

In this subsection, the concept of the GP techniques is introduced and how the GP was utilized in this work is described. The GP is a Gaussian distribution over functions, described as:

(4) $$f_{GP}∼GP\left(m\left(x\right),k\left(x^{\prime },x\right)\right)$$

where m(x) is the mean function and $k(x^{\prime },x)$ is the covariance function between $x^{\prime }$ and x. A constant mean function m(x)=c where *c* is one of hyperparameters to be optimized is used and the following squared exponential covariance function [16] is adopted in this study.

(5) $$k\left(x^{\prime },x\right)=\sigma_{f}^{2}{\left(\frac{a}{b}\right)}^{\frac{n}{2}}\exp\left(\frac{||x-x^{\prime }{\left|\right|}^{2}}{b}\right),$$

where $a=2l\left(x\right)l\left(x^{\prime }\right)$, $b=2l^{2}\left(x\right)+l^{2}\left(x^{\prime }\right)$ and *n* is a number of variables being correlated. l(·) represents the spatially-varying length scale hyperparameter and $\sigma_{f}$ is another hyperparameter to be optimized, which determines the magnitude of covariance. A training set with $N_{t}$ observations is expressed as $D=\left\{\left(x_{n},y_{n}\right)|n=1,\cdots ,N_{t}\right\}=\left\{X,y\right\}$ where X is a set of input vectors consisting of the position of the UAV and ground nodes and y is a set of measured signal strength values from ground nodes. The GP model is evaluated by marginal likelihood as:

(6) $$\begin{array}{cc} L(\theta ) & =\log\left(y|X,\theta \right)=-\frac{1}{2}\log\left|C_{n}\right|\\ & -\frac{1}{2}{\left(y-m\left(x\right)\right)}^{T}{\left(C_{n}\right)}^{-1}\left(y-m\left(x\right)\right)-\frac{N_{t}}{2}\log\left(2\pi \right) \end{array}$$

where hyperparameters θ are the parameters to be trained to fit the mean and covariance functions to the training data. To be more specific, hyperparameters are used to compute the value of m(x) and $C_{n}$ in Equation (6). $C_{n}=\Sigma +{\bar{\sigma }}_{n}^{2}I_{N_{t}}$, in which Σ denotes a set of covariance functions of $N_{t}\times N_{t}$ size with entries $k_{ij}=k\left(x_{i},x_{j}\right)$ for $i,j=1,\ldots ,N_{t}$. ${\bar{\sigma }}_{n}^{2}$ is the hyperparameter accounting for noisy data. To obtain the trained GP, the hyperparameters should be optimized by maximizing the marginal likelihood. Given the training set D and the covariance function with the trained hyperparameters, the mean and variance at an arbitrary test position $x^{*}$ are computed as:

(7) $$\begin{array}{cc} \mu_{p}\left(x^{*}\right) & =m\left(x^{*}\right)+k{\left(x,x^{*}\right)}^{T}(C_{n}^{-1}\left(y-m\left(x^{*}\right)\right) \end{array}$$

(8) $$\begin{array}{cc} \sigma_{p}^{2}\left(x^{*}\right) & =k\left(x^{*},x^{*}\right)-k{\left(x,x^{*}\right)}^{T}{\left(C_{n}\right)}^{-1}k\left(x,x^{*}\right). \end{array}$$

where $m(x^{*})$ is the mean function value at the test position $x^{*}$ and $k(x,x^{*})$ is a set of covariance functions of size $N_{t}\times 1$ between all training points collected so far and the test position $x^{*}$. Here, $\mu_{p}\left(x^{*}\right)$ represents the predicted signal strength at the test position $x^{*}$ and $\sigma_{p}^{2}\left(x^{*}\right)$ represents how accurate the GP prediction $\mu_{p}\left(x^{*}\right)$ is at that position. After the relay UAV scans the area, the collected RSSI values during the scan flight and the corresponding positions of the UAV are used to train the GP; this creates the communication map of the area (i.e., predicted RSSI values with respect to arbitrary UAV positions for a given region of interest). This GP-based channel prediction has the following benefits compared with the model-based approach in relay missions:

- GP prediction requires relatively less preliminary effort. The GP-based approach autonomously computes the optimal hyperparameters of the GP model using collected measurements while the model-based approach should go through the exhausting procedure described in Section 3.1.
- GP prediction does not use a 3D map of the city. However, the model-based approach requires a map of the city to provide the building obstruction information used for Equation (3).
- The GP approach is able to deal with environmental changes by quickly re-scanning the city. On the contrary, it is hard to obtain relevant parameters in the model-based approach when some changes occur in the environment in terms of wireless communication.

However, in general, the GP method suffers from growing computational burden as the number of observations increases; the computational complexity of the GP is $O(N_{t}^{3})$ where $N_{t}$ is the size of training data (i.e., the number of measurements). The feasibility of the GP method in real time is verified in the following sections.

## 4. Experimental Setup

This section introduces experimental setup including artificial urban environments, mesh network protocol, ground nodes and an aerial relay UAV. Due to the limitations of experiments in real urban environments such as flight restriction and experiment repeatability, a down-scaled indoor urban environment was employed, which reserved similar communication characteristics to outdoor urban environments. The 802.11 s mesh protocol was adopted for the wireless communication through rt5870 Wi-Fi chipsets in the experiments. Comparison amongst several mesh network protocols was provided to justify why the 802.11 s mesh protocol was used. Two Turtlebot UGVs for the ground nodes and a quadrotor UAV for the aerial relay node were used in the robot operating system (ROS) environment. The localization of the UAV was obtained via the Vicon motion capture system. A separate laptop was used for the GP-based channel prediction algorithm. As mentioned above, water containers were used for obstructions of buildings.

### 4.1. Artificial Indoor Urban Environment

Performing experiments involving communication relay UAVs in a real urban environment is challenging. Regulations heavily restrict flights in an urban environment. Although it could be possible to utilize military or firefighter’s urban training grounds, those environments would be limited in repeatability. Indoor experiments can address those limitations as the experiment can be repeated as many times as necessary to obtain meaningful results.

However, the use of indoor areas poses some challenges of effectively emulating effects on communication signal transmission in the urban environment. First, due to the limited size of the buildings in the scaled environment, typical building materials such as bricks and concrete are not thick enough to attenuate signal significantly. This is particularly an issue considering the low cost Wi-Fi dongles used in this experiment, which are even less likely to detect small differences in signal strength. The second challenge is making the indoor urban environment easily adaptable to extensively test the performance of the GP-based prediction.

**Figure 6:**
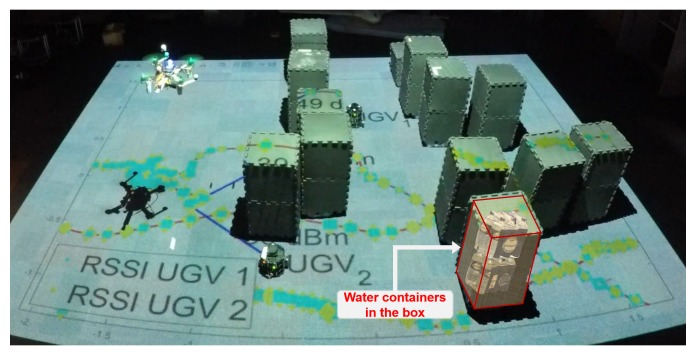
A snapshot of an indoor flight experiment in an artificial urban environment where there are water containers inside boxes representing buildings.

To address those challenges, the use of water as a material for the building is proposed. Water is excellent at absorbing 2.4 GHz wireless signal even with a small obstruction [17]. With the significant signal strength change when the line-of-sight between nodes is obstructed by the building, typical Wi-Fi dongles are capable of detecting the difference easily. Moreover, water containers can be easily moved and stacked to create different city layouts, as shown in Figure 6. In this work, an artificial urban environment of 5 m by 5 m size was considered.

### 4.2. Mesh Networks Protocols

To allow data routing within the mesh topology, an appropriate protocol has to be chosen. One of important requirements is the compatibility with the robot operating system (ROS) as all devices were running on ROS framework in this experiment. The second requirement is the ability to send data quickly and reliably to facilitate the position data transfer between the Vicon motion tracking system and the relay UAV. This delay cannot be longer than 0.2 s; otherwise, the autopilot (Pixhawk used in this experiment) assumes that data are too old and starts an emergency descent procedure. Note that this delay is not only communication delay but also time needed for the Vicon motion capture system to generate the positional data and for ROS to translate to/from mavlink messages to/from appropriate message types. To fulfill those requirements, three protocols were considered: BATMAN Adv. [18], IEEE 802.11 s [19] and IEEE 802.15.4 ZigBee [20].

**BATMAN**. A better approach to mobile ad-hoc network (BATMAN) is designed as a low computational complexity with a distributed networking protocol. In this approach, each node on the network only holds information about neighbors and the general direction to the destination node, rather than full routing information. With limited knowledge, each node can determine the sub-optimal route quickly. BATMAN is well-documented and is capable of working with the ROS.

**802.11 s**. This protocol is a standard mesh networking developed by IEEE. It has several distinct features compared to other approaches. First, each node on the network can act as a mesh station, mesh access point or mesh portal. A mesh station is used to connect 802.11 s to other 802.11 based networks. Mesh access point can forward and receive packets within the 802.11 s network. A mesh portal has a very similar function to an access point but provides services to other non-802.11 networks such as 802.3. For data transfer, the following procedure is obeyed. Initially, the path request from the origin node to the destination node is sent out. Each node adds either its own ID in sequence and forwards it to its neighbors, or if it knows the route to the target node, it simply fills the rest of the table. Once the full destination node is reached, the optimal route is determined, and a route table with confirmation of destination is sent back to the origin. The route table is cached for some pre-specified amount of time for future usage.

**802.15.4**. This protocol is a mesh implementation relying on ZigBee infrastructure. ZigBee is a small and low-powered radio commonly found in UAV applications due to its weight and size. In 802.15.4, one of the nodes is called the coordinator. The coordinator is responsible for holding information about routes and make them available on request from any of the ground nodes. With this single node holding all routing information, routing can be performed almost optimally.

A comparison between the performance of protocols needs to be performed in order to choose an appropriate networking protocol. There does not exist a direct comparison amongst the three protocols in the literature to the best our knowledge. Thus, the comparison was conducted between BATMAN against 802.11 s based on the work in [21] first. In this work, it was shown that 802.11 s has much lower throughput than BATMAN. The 802.11 s standard is using the 802.11g standard as the underlying architecture, as opposed to the 802.11n standard used in BATMAN. 802.11g has a maximum throughput of 54 Mbps while 802.11n has a throughput of 300 Mbps. On the other hand, 802.11 s showed an advantage in two aspects: reduced latency and increased data delivery reliability. The suboptimal routing methodology in BATMAN means that many packets are simply lost or take a very long route. It is worth reiterating that one of the essential requirements for our purpose is the reliability of data transfer in the network to facilitate fast transmission of position data to the UAV. Moreover, the high bandwidth is not of primary concern as data size used in this experiment is small. Thus, 802.11 s could be regarded as the better solution for this problem than BATMAN considering relatively enhanced data delivery reliability and reduced latency.

With the 802.11 s standard being better than BATMAN, it only remains to compare 802.11 s with 802.15.4. The choice between those two standards can be made using the second requirement criterion, compatibility with the ROS. 802.11 s is compatible with the ROS “out of the box” while 802.15.4 requires an external package such as rosserial xbee [22]. Encoding and decoding data to and from the ROS would likely introduce delays in data transfer. This can result in slow transmission of position data. Consequently, the 802.11 s standard is adopted for the mesh network protocol in this study.

### 4.3. Ground Node

**Figure 7:**
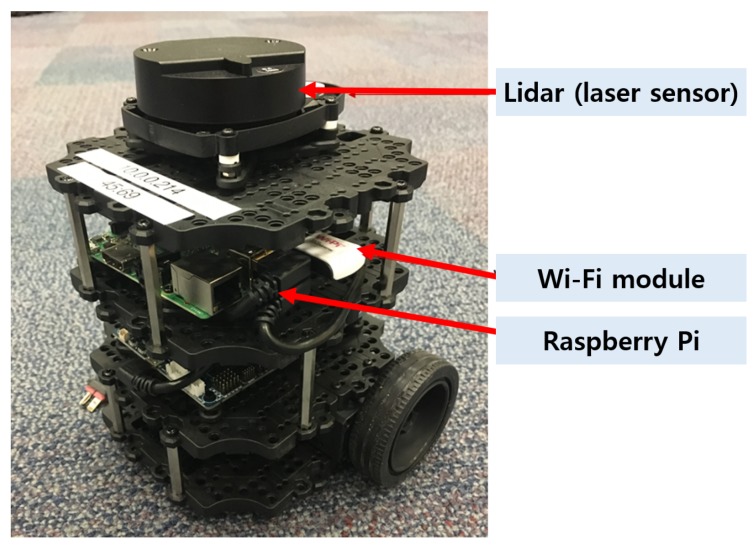
Overview of the Turtlebot 3 UGV used in this experiment with important components highlighted.

For the ground nodes, the Turtlebot 3 Burger UGV was utilized, as shown in Figure 7. The Turtlebot is equipped with a Raspberry Pi, an open CR board and a Lidar.

***Raspberry Pi***. It is a main onboard computer of the Turtlebot which runs on Ubuntu 16.04 with ROS Kinetic. An external Wi-Fi dongle is attached for the mesh network connections. It is compatible with 802.11 s. Although a Raspberry Pi has a built-in Wi-Fi module, its drivers are incompatible with 802.11 s. In the Turtlebot, the computing board is responsible for running the Turtlebot packages. Raspberry Pi connects to the ROS node with an onboard OpenCR controller.

***OpenCR***. It is a controller board for Turtlebots. The OpenCR board is responsible for translating commands from the onboard computer to motors and power distribution. The board is based on Arduino microcontroller and uses a serial port to communicate with the Raspberry Pi.

### 4.4. Aerial Relay Vehicle

**Figure 8:**
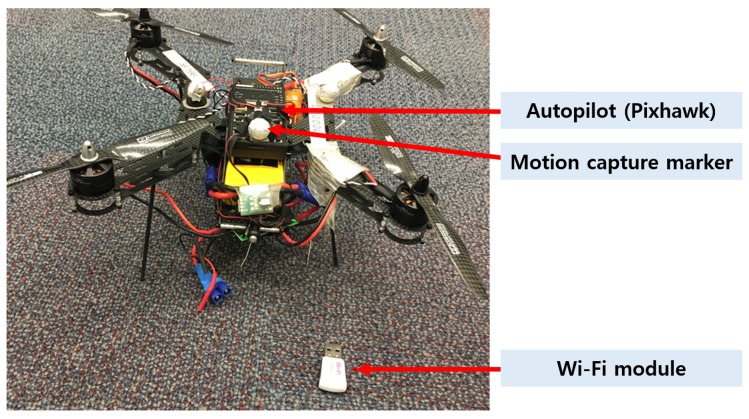
Overview of the quad-rotor UAV used in this experiment with important components highlighted.

**Figure 9:**
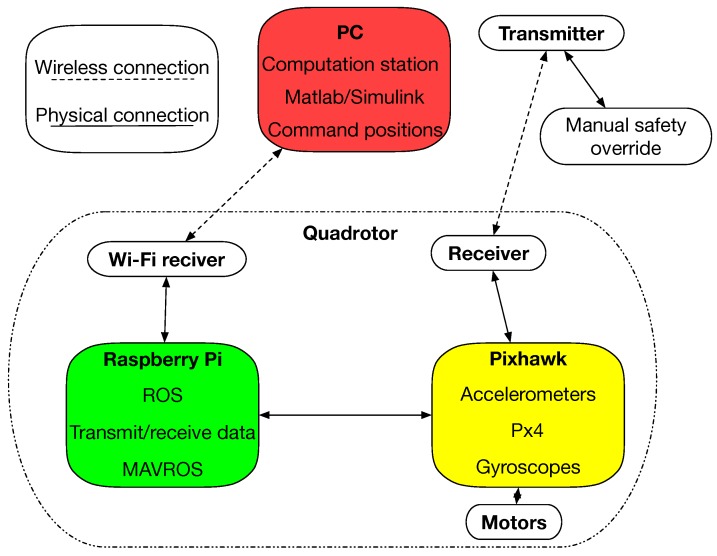
System overview for the aerial relay vehicle.

In this experiment, a custom built quad copter UAV was used, as shown in Figure 8. It is a standard F300 size frame with large 3 cell 5000 mAh battery for extended endurance. A more specific component breakdown of both the hardware and the software is shown in Figure 9.

***Pixhawk***. The Pixhawk is a commercial off-the-shelf autopilot and popular in the small UAV field. In terms of hardware, the Pixhawk provides a broad range of sensors including a 6-DOF IMU, GPS and barometer. On the software side, the PX4 flight stack is used. With PX4 and the MAVlink protocol, most of the PX4 data inputs and outputs are available over the serial port. MAVROS interprets these on the Raspberry Pi.

**Figure 10:**
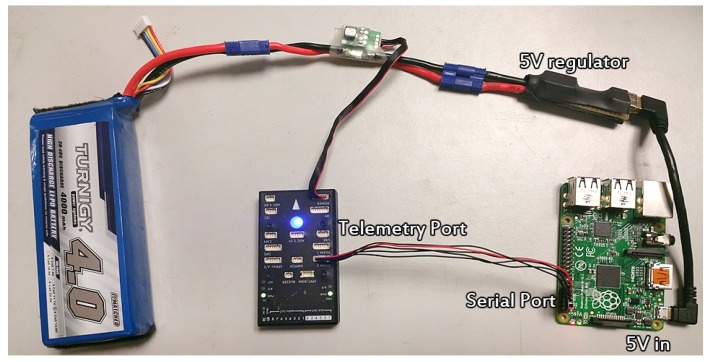
Hardware overview of common ROS/Autopilot system components.

***Raspberry Pi***. A popular credit card-sized computer running Ubuntu 16.04 and ROS Kinetic. The Raspberry Pi on board the UAV has two main purposes. Firstly, it is used to connect quad-rotor UAV with the rest of the 802.11 s network. Secondly, it acts as an interpreter and relay for messages between various sources and the autopilot. The Pixhawk communicates with a Raspberry Pi through a serial port, as shown in Figure 10.

***MAVROS*** [23] This is a ROS package and bridges between ROS messages and the MAVlink standard used on autopilots. MAVROS topics include transmitter output, position in a global and local frame, aircraft attitude and speed and many more. To control the UAV from the external computer, a facility called “offboard mode” is utilized. Attitude, position and velocity commands can be sent to Pixhawk through the offboard mode.

***Wi-Fi Receiver*** The Wi-Fi interface is used to provide network connectivity between the quad-rotor UAV and the rest of the system. The adapter used here is based on the rt5780 chipset to ensure the best compatibility with the 802.11 s standard. For more details regarding the system, the reader is referred to the recently-published ROS overview [24].

## 5. Experimental Results

Indoor flight experiments in various environments were carried out to validate the performance of the GP-based channel prediction method. The first experiment (Case I) was performed with one UGV and one relay UAV flying a back-and-forth scan pattern in an open space at a constant height. In the second experiment (Case II), two UGVs were used. For the third experiment (Case III), one building was added between two UGVs in order to compare the performance of the GP and model-based prediction on a slightly more complex scenario. Finally, randomly-generated city environments (Case IV) were tested.

Experiments were performed with the following procedure. First, the relay UAV scanned the city at a 1 m height by collecting the signal strength data. Then, the discretized communication strength grid map for each UGV was computed by GP-based prediction. Finally, the UAV stayed at the optimal position for 15 s to collect the actual RSSI data. The optimal position was determined by finding the position having the maximum sum of the signal strength between the UAV and all ground nodes.

### 5.1. GP Computation Time

About 1800 measurements were obtained during scan flight in each test; half of the collected data were used to train the GP model and the rest were used for error analysis. The computation time for the GP with two UGVs was 11.4 s on average on the Intel Core i7-5775 processor using the GPML toolbox written in Matlab [25]. The computational time included both optimizing the GP hyperparameters and generating the discretized communication strength grid map for the entire indoor environment where the distance between grid points was set for 15 cm. This implies that the GP-based prediction approach could be used in real-time relay applications. Besides, the computation time could be significantly improved if implemented in faster programming languages such as C/C++.

### 5.2. Case I: Single UGV in an Open Space

**Figure 11:**
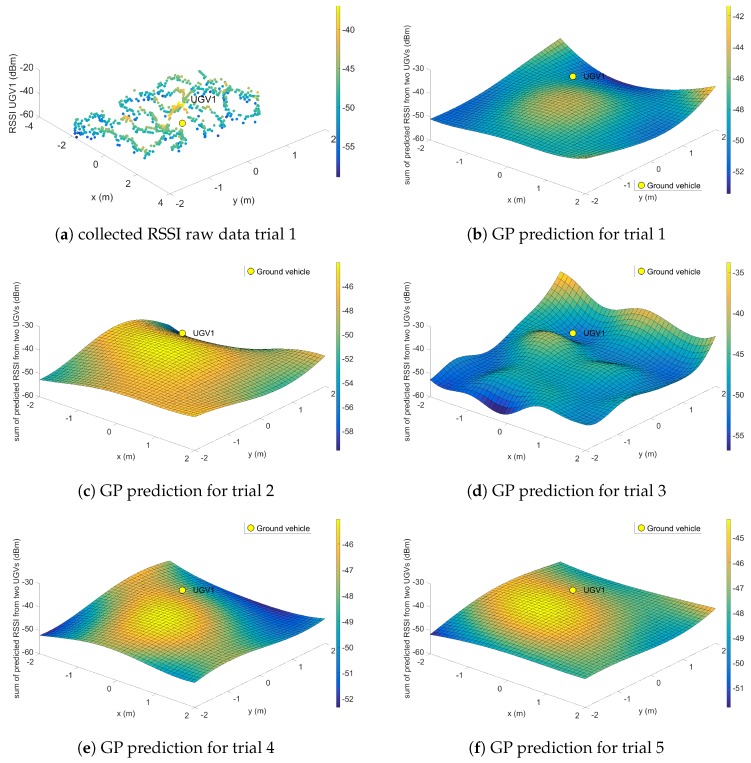
Experiment result for Case I.

For the single UGV experiment, the Turtlebot UGV was placed in the centre of the room so that the USB Wi-Fi dongle on the UGV was at the origin in Cartesian coordinates. The relay UAV performed the pre-planned pattern flight to collect the RSSI data, as shown in Figure 11a. Comparing experimental trials in Figure 11, it can be seen that, across the trials, the maximum RSSI prediction occurred on or close to the origin. This was expected behavior as the signal strength should be the strongest near the Wi-Fi dongle of the UGV. It can be noted that, sometimes in the UAV boundary area, the GP predicted a high signal strength value. This was caused by a few random high RSSI values on the edges during the back-and-forth scan pattern. This could also be caused by reflections from the metal wall of the building.

### 5.3. Case II: Two UGVs in an Open Space

Two UGVs in an open space (i.e., without any buildings) were used in the experiment. The main purpose of this experiment was to compare the communication relay performance of the GP-based prediction against the empirical communication model in a simple environment.

**Figure 12:**
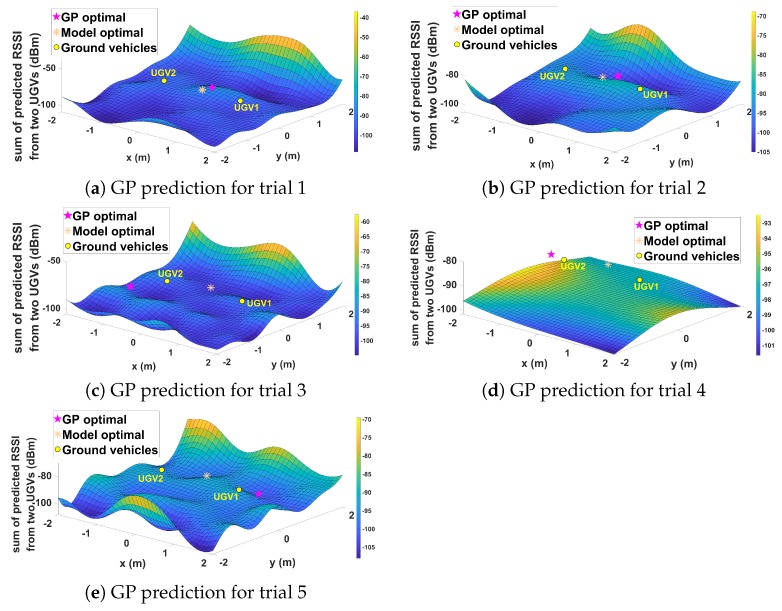
Experiment results for Case II.

Figure 12 shows the communication map predicted by the GP method and predicted optimal relay positions of GP and model-based approaches for each trial. For model-based communication prediction, the optimal relay position was apparently in the middle between two ground nodes. For GP-based prediction, the optimal position varies, as shown in Figure 12. This variation was due to the error of GP prediction and dynamic characteristic of the wireless communication environment at the time of the experiment. Table 1 provides actual signal strength values averaged for 15 s from two ground nodes at predicted optimal positions from GP and model-based approaches. On average, the GP had slightly better performance compared to the model-based approach. It is worth e noting that, for Trial 3, the GP performance was better than that of the model-based approach despite the optimal position not being in the middle of two ground nodes. This clearly showed the benefit of learning-based GP prediction, which is the ability to adapt to dynamic and unknown communication environments.

### 5.4. Case III: Two UGVs with One Building

**Figure 13:**
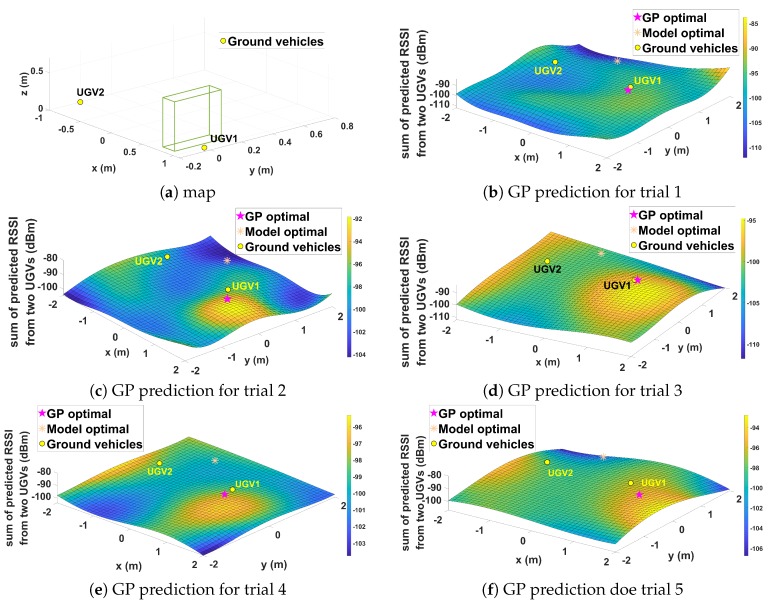
Experiment results for Case III.

In this case, one building was placed close to one of the ground nodes to obstruct the line-of-sight between them, as shown in Figure 13a. In Figure 13, it can be noted that the predicted optimal positions of the UAV were quite different between two prediction methods. However, based on Table 2, their performances were similar on average with a slight advantage of the GP-based approach. This is a promising result, implying that, in a realistic setting, the GP can have performance similar to that of a good empirical model. In fact, the GP-based method is more attractive than the model-based method in a practical manner: the GP approach does not require a priori knowledge such as 3D city map and communication model parameters required for model-based approach.

### 5.5. Case IV: Two UGVs in Complex Cities

**Figure 14:**
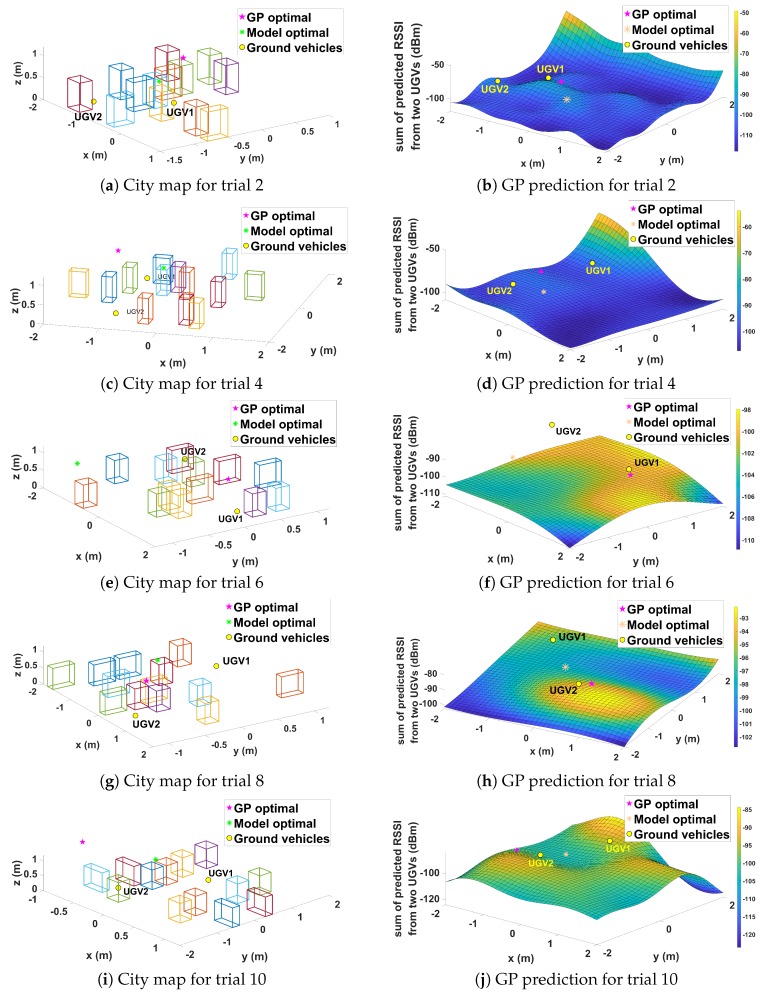
Experiment results for Case IV.

Lastly, ten randomly-generated complex cities were introduced. The random cities consisted of buildings with the same height but different positions and rotations for each trial. These random cities were used to show the robustness of the GP-based prediction in different scenarios. Due to the space limitation, five of the ten trials are displayed. Comparing the results in Figure 14, it can be noted that best positions varied widely between the model and GP-based approaches. Table 3 also shows significantly different performance results across trials. On average, the GP method had a similar performance to the communication model-based approach with a slight advantage; this is consistent with earlier results. It can be noted that, in some scenarios, the performance of the model approach was better than the GP approach. In a complex scenario such as this, with many water containers inside the boxes, the stochastic nature of wireless communication was particularly prominent, which means that it is possible for the GP to make erroneous prediction. However, the GP approach can still be considered useful as it does not require a priori information about the environment.

**Figure 15:**
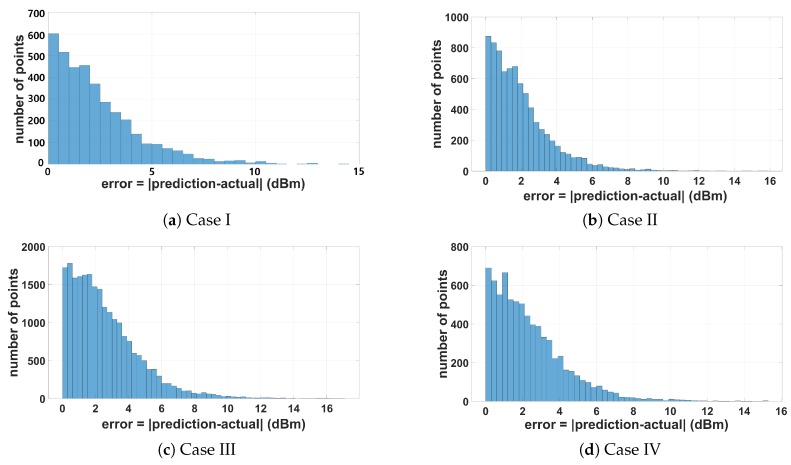
Error histogram averaged over multiple runs.

The error in prediction is displayed in Figure 15. Most of the errors are less than 5 dBm. The relatively high error of very few data points can be explained by smoothing tendencies of the GP. The GP prediction will never entirely match the actual signal strength close to the Wi-Fi dongle of the UGV. In GP, such a high reading close to the Wi-Fi dongle is smoothed out by the relatively low signal strength reading nearby. This is one of the well-known limitations of the GP and could be addressed by the use of the Gaussian process mixture techniques described in [26]; this remains as future work. A movie clip for an indoor experiment in this complex urban environment can be found at: https://youtu.be/rFXolMM6CNA.

## 6. Conclusions Future Work

This study conducted flight experiments to evaluate the performance of Gaussian Process-based channel prediction in an urban environment. An artificial urban environment was introduced to alleviate the limitations from the flight restrictions in outdoor urban environments. Several experiments were performed for four cases with the different number of ground nodes and buildings: (i) one UGV in an open space; (ii) two UGVs in an open space; (iii) two UGVs with one building; and (iv) two UGVs in complex city environments. The fact that GP computation took about 10 s to train with about 900 training points and generate the discretized communication grid map shows the feasibility of the GP method in real time. The performance of GP-based prediction was consistent across all experiments and outperformed the model-based approach. Outdoor experimental validation in more realistic settings will be followed as future work.

## Figures and Tables

**Table 1:** Actual signal strength value (RSSI) at the predicted optimal position for Case II.

Trial	GP (dBm)	Model (dBm)
1	−91.15	−92.79
2	−86.44	−92.87
3	−95.46	−99.66
4	−96.53	−99.33
5	−94.93	−100.86
Mean	−92.90	−97.10

**Table 2:** Actual signal strength value (RSSI) at the predicted optimal position for Case III.

Trial	GP (dBm)	Model (dBm)
1	−95.90	−98.91
2	−96.74	−95.33
3	−93.4	−98.5
4	−96.08	−97.94
5	−98.06	−95.97
Mean	−96.04	−97.34

**Table 3:** Actual signal strength values (RSSI) at the predicted optimal position for Case IV.

Trial	GP (dBm)	Model (dBm)
1	−101.33	−97.20
2	−94.70	−111.62
3	−91.27	−98.30
4	−99.57	−99.09
5	−98.51	−100.63
6	−97.81	−103.98
7	−95.56	−92.68
8	−92.57	−94.73
9	−95.26	−93.23
10	−101.48	−94.17
Mean	−97.10	−98.56
